# Neutrophil extracellular traps contribute significantly to vascular dysfunction in sepsis

**DOI:** 10.3389/fphar.2026.1815355

**Published:** 2026-06-17

**Authors:** Pingping Zhang, Yajuan An, Haizhao Liu, Dongqiang Wang

**Affiliations:** 1 Department of Integration of Traditional Chinese and Western Medicine, First Central Hospital Affiliated to Nankai University, Tianjin First Central Hospital, Tianjin, China; 2 Tianjin Medical University, Tianjin, China

**Keywords:** endothelial injury, neutrophil extracellular traps, neutrophils, sepsis, therapeutic strategies, vascular dysfunction

## Abstract

Sepsis is a systemic inflammatory condition triggered by severe infection, frequently resulting in multi-organ dysfunction. Vascular dysfunction, as its core pathological mechanism, involves a vicious cycle of inflammation, coagulation, and endothelial injury. The mechanism of vascular damage caused by sepsis is widely studied, and neutrophils play a significant role in this process. Neutrophil extracellular traps (NETs) are an important mechanism. While NETs are designed to entrap pathogens, their excessive formation or impaired degradation directly drives vascular injury. NETs contribute to the exacerbation of vascular dysfunction through mechanisms including the induction of endothelial injury, the promotion of coagulation abnormalities, and the enhancement of vascular permeability. Nonetheless, the precise signaling pathways and regulatory networks governing these processes remain incompletely understood. This article seeks to systematically review the fundamental mechanisms through which NETs contribute to sepsis-associated vascular dysfunction, in addition to evaluating their potential utility as biomarkers and therapeutic targets. This review seeks to elucidate novel insights into the mechanisms underpinning vascular dysfunction in sepsis and associated clinical interventions, thereby contributing a theoretical foundation for the development of targeted therapeutic strategies to mitigate related pathophysiological damage.

## 1 Introduction

Sepsis is a systemic inflammatory response syndrome caused by infection, leading to life-threatening organ dysfunction and tissue damage (Guo et al., 2023; Bauer et al., 2018). Its pathophysiological process is characterized by uncontrolled host immune responses, widespread vascular endothelial injury, and coagulation dysfunction, marked by hypotension and reduced vasoconstrictor responsiveness (Barp et al., 2020). The core of Sepsis lies in the convergence of inflammation, coagulation, and endothelial injury, forming a self-amplifying thrombotic inflammatory network (Nishibori, 2022; Iba et al., 2025). This dysregulated host response leads to microcirculatory dysfunction, with endothelial cell injury being the primary factor in microcirculatory problems (Pan et al., 2026). In the complex pathophysiological process of sepsis, the central role of neutrophils and their released neutrophil extracellular traps in vascular injury mechanisms has gained increasing attention in recent years (Chen et al., 2022). Neutrophil extracellular traps (NETs) have emerged as dual-edged players in sepsis pathophysiology. While NETs play a crucial role in pathogen elimination by capturing and neutralizing microorganisms, their excessive formation or inadequate clearance can lead to endothelial cell damage, activation of the coagulation cascade, and the release of inflammatory mediators, ultimately exacerbating vascular dysfunction. NETs not only directly attack endothelial cells through their inherent cytotoxic components but also serve as scaffolds for immune thrombosis, tightly linking inflammation, coagulation, and endothelial dysfunction into a self-amplifying vicious cycle (Iba et al., 2025).

Consequently, a comprehensive understanding of the precise mechanisms through which NETs induce vascular damage in sepsis is essential for the advancement of targeted therapeutic interventions. Current research suggests that NET release is modulated by various signaling pathways. For example, the triggering receptor expressed on myeloid cells-1 (TREM-1) enhances Toll-like receptor-4 (TLR4)-mediated inflammatory responses, thereby promoting NET release in both human and murine neutrophils. Inhibition of TREM-1, either pharmacologically or through genetic knockout, has been shown to reduce NETosis in both *in vitro* and *in vivo* models of experimental septic shock (Boufenzer et al., 2021). Moreover, glycolytic metabolism involving 6-phosphofructo-2-kinase/fructose-2,6-bisphosphatase 3 (PFKFB3) facilitates sepsis-induced NET formation via peptidylarginine deiminase 4 (PAD4)-mediated chromatin decondensation, which subsequently disrupts endothelial barrier function (Yin et al., 2026). In cases of sepsis-induced acute lung injury, NETs activate the coagulation cascade in endothelial cells through a stimulator of interferon genes (STING)-dependent mechanism by activating the interferon-inducible gene activator pathway (Zhu S. et al., 2023). Additionally, NET accumulation hinders pulmonary endothelial cell proliferation and vascular repair by inhibiting the Polo-like kinase 1 signaling pathway, leading to G2/M phase cell cycle arrest (Zhu et al., 2025). This review will systematically explore these mechanisms and their implications for therapeutic development.

## 2 The formation mechanism and regulation of NETs

### 2.1 NET production

Neutrophils constitute approximately 70% of human white blood cells and serve as the first line of defense against invading pathogens. Mature neutrophils, as integral elements of the innate immune system, become activated upon stimulation and engage in phagocytosis, degranulation, and the formation of NETs in response to pathogenic threats. NETs play a crucial role in immune defense by targeting and eliminating bacteria, viruses, and fungi (Narasaraju et al., 2011; Saitoh et al., 2012; Urban et al., 2006; Islam and Takeyama, 2023; Brinkmann et al., 2004). NETs are extracellular structures made of DNA, histones, neutrophil granule proteins, and proteolytic enzymes (Brinkmann et al., 2004; Brinkmann and Zychlinsky, 2007). They trap and eliminate pathogens via NETosis, a process that often leads to neutrophil death (Vorobjeva and Chernyak, 2020). This mechanism, integral to the innate immune response, can yield both advantageous and detrimental effects (Meier et al., 2024; Brinkmann and Zychlinsky, 2012; Papayannopoulos et al., 2010). NETs inevitably play a beneficial role in host protection (Huang et al., 2020).

**FIGURE 1:**
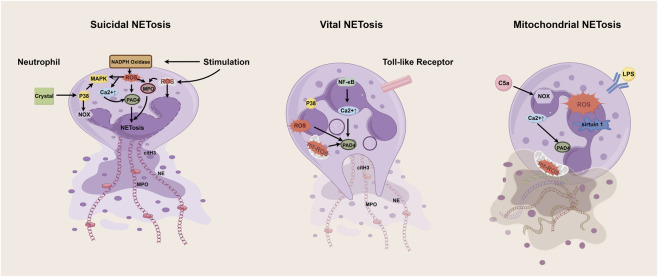
In NETosis, external stimuli activate membrane receptors, triggering the release of NETs via various internal pathways, including Suicidal, Vital, and Mitochondrial NETosis.Suicidal NETosis triggers diverse signaling pathways when stimulated by bacteria, chemicals, and immune complexes, resulting in morphological alterations in neutrophils. These encompass changes in nuclear structure, chromatin disorganization, enhanced nuclear envelope and granule membrane permeability, and the extracellular release of DNA and granules. Unlike suicidal NETosis, Vital NETosis occurs independently of Nox and does not necessitate ROS formation. Furthermore, neutrophils undergoing this mechanism type can survive activation with intact nuclei, membranes, and plasma membranes, retaining chemotactic and phagocytic activity. Post-activation, vesicle-released granular material is detectable. Mitochondrial NETosis generates nets consisting of mitochondrial DNA instead of nuclear DNA. Like suicidal NETosis, it depends on ROS; however, it does not cause membrane rupture or lead to cell death.

Neutrophils, when activated by C-X-C motif chemokine ligand 8(CXCL8), phorbol 12-myristate 13-acetate (PMA), or Lipopolysaccharide (LPS), release denatured chromatin and granule-derived proteins, including neutrophil elastase, myeloperoxidase, cathepsin G, lactoferrin, and gelatinase. These components collectively form an extracellular reticular network that effectively traps and kills bacteria. The release of NETs represents a distinctive defense strategy utilized by neutrophils in reaction to pathogenic threats or particular inflammatory stimuli. The formation of NETs is not confined to a singular pathway; rather, it transpires through diverse mechanisms, predominantly encompassing suicidal NETosis, vital NETosis, and mitochondrial NETosis. Additionally, there are NETosis associated with apoptosis, NETosis related to autophagy, and cross-cell NETosis triggered by interactions between neutrophils and other cells (Brinkmann et al., 2004) (Figure 1).

#### 2.1.1 Suicidal NETosis

Suicidal NETosis refers to the release of NETs through a sequential series of cellular events, accompanied by neutrophil lytic death, and is dependent on the presence of reactive oxygen species (ROS). Suicidal NETosis occurs when neutrophils are activated by different stimuli. Multiple pathogens, such as *Staphylococcus aureus* and Cryptosporidium parvum, can induce suicidal NETosis (Monteith et al., 2021; Hasheminasab et al., 2022). Surface receptors, including G protein-coupled receptors, Fcγ receptor 7, and complement receptors, perceive these stimuli (Fang et al., 2021; van der Velden et al., 2024). This receptor participates in the release of calcium ions from the endoplasmic reticulum. Calcium influx subsequently triggers downstream kinase-mediated signal transduction. Activation of protein kinase C, p38 mitogen-activated protein kinase (MAPK), and Rac signaling can induce NADPH oxidase or mitochondrial respiration to produce ROS (Zhu Y. P. et al., 2023; Li et al., 2022; Yu et al., 2024; Powell et al., 2022). In the classical suicidal NETosis pathway, the production of ROS by NADPH oxidase is the key initiation step of the cascade reaction (Cao et al., 2022). Elevated ROS levels trigger the release of neutrophil elastase (NE) and myeloperoxidase (MPO) from azurophil granules into the cell nucleus, facilitating chromatin decondensation via proteolytic histone cleavage and charge neutralization. Concurrently, ROS synergistically interacts with intracellular calcium to activate PAD4, converting arginine residues on histones into citrulline, thereby inducing chromatin decondensation. Condensed chromatin disperses throughout the cytoplasm, integrating with cytoplasmic and granular proteins. In later stages, cytoplasmic NETs bind and degrade the cytoskeleton to block phagocytic pathways. Ultimately, Gasdermin D (GSDMD) is cleaved by serine proteases, including neutrophil elastase (NE), resulting in the formation of pores in the cell membrane. This process culminates in the disruption of the cell membrane integrity, facilitating the extracellular release of chromatin. The released chromatin, which encompasses MPO, NE, and citrullinated histones, contributes to the formation of NETs (Sollberger et al., 2018).

#### 2.1.2 Vital NETosis

In contrast to suicidal NETosis, which culminates in cell death, vital NETosis represents a mechanism of NETs release that does not precipitate immediate neutrophil demise. During this process, upon appropriate stimulation, neutrophils employ vesicular transport to encapsulate nuclear DNA and subsequently expel it extracellularly. While the nuclear membrane may undergo rupture, the integrity of the plasma membrane is preserved, thereby enabling the neutrophils to maintain their phagocytic and chemotactic functions (Li et al., 2025). Vital NETosis generally does not depend on NADPH oxidase-mediated ROS bursts; however, it remains reliant on PAD4 activity to facilitate histone citrullination and chromatin decondensation (Pruchniak et al., 2025; Wang et al., 2009; Li et al., 2010). It operates with increased rapidity, generally facilitating the release of NETs within 5–15 min following neutrophil activation, without compromising neutrophil functionality, thus offering sustained defense during the initial phases of infection (Pilsczek et al., 2010; Yan et al., 2025).

#### 2.1.3 Mitochondrial NETosis

Mitochondrial NETosis represents a distinct mechanism of NET formation that has garnered significant attention in recent years. In contrast to the classical pathway, which depends on NADPH oxidase to facilitate the release of nuclear DNA, mitochondrial NETosis predominantly involves the release of mitochondrial DNA as the structural framework for NETs, while nuclear DNA is not released. Upon activation by Granulocyte-macrophage colony-stimulating factor (GM-CSF) and subsequent stimulation with complement factor 5a/LPS, neutrophils release mitochondrial DNA into the extracellular space in a ROS-dependent manner, with the DNA being encapsulated by granule proteins. Mitochondrial complexes I and III, in addition to NADPH oxidase, contribute to neutrophil bactericidal activity. Furthermore, the deacetylase transcription factor sirtuin 1 promotes the opening of the mitochondrial permeability transition pore channel for the externalization process of mitochondrial NETs (Dunham-Snary et al., 2022; Frost et al., 2022). Neutrophils release NETs when exposed to various stimuli. PAD4 facilitates histone citrullination, leading to chromatin depolymerization (Lewis et al., 2015). Activated NADPH oxidase generates reactive oxygen species. ROS activate PAD4, promoting chromatin decondensation by modifying arginine residues on histones H3 and H4 (Wang et al., 2004). This process leads to the separation of histone H1 and heterochromatin protein 1b from the nucleosome structure (Leshner et al., 2012). Chromatin decompaction leads to nuclear membrane disruption, releasing nucleosomes into the cytoplasm to form NETs. Disrupting NET structure can inhibit the immune response activated by them (Clark et al., 2007; Dąbrowska et al., 2016).

NETs can be generated in different areas of the body and are released wherever neutrophils exist. During sepsis, neutrophils are found in various organs, including the lungs, liver, intestines, kidneys, heart, and throughout the bloodstream. They engage with platelets, endothelial cells, and the complement system to facilitate NETs formation and immune thrombosis (Chen et al., 2021). Research indicates that under pathological conditions such as sepsis, platelets can induce NETosis by releasing factors, including high-mobility group box protein B1 (HMGB1), thereby promoting mitochondrial ROS production and autophagy (Wu et al., 2025). Moreover, the neutrophil elastase that is released aids in chromatin depolymerization and histone breakdown. The breakdown of the nuclear and cytoplasmic membranes ultimately leads to NET release (Metzler et al., 2014). This lysis pathway can result in “cellular suicide” via NETosis or function as a crucial non-solubilizing mechanism to trap and dissolve pathogens (Pilsczek et al., 2010), including bacteria, fungi, parasites, and viruses (Papayannopoulos, 2018). Although NETs can protect the host from microorganisms, excessive NETosis can be detrimental to the host’s tissues. Recent *in vitro* and animal studies suggest that NETs play a crucial role in the development of certain autoimmune and autoinflammatory diseases, as well as specific sepsis types, thereby elevating morbidity and mortality rates (Mutua and Gershwin, 2021).

The three pathways exhibit significant differences in induction signaling, kinetic characteristics, cell fate, and molecular mechanisms. Suicidal NETosis represents the classical model of NETosis, characterized by its reliance on NADPH oxidase to produce ROS, which subsequently induce nuclear chromatin depolymerization and nuclear membrane rupture, ultimately resulting in cell death. In contrast, vital NETosis involves the direct activation of PAD4 through calcium influx, leading to the release of DNA via vesicles while preserving cell viability. Mitochondrial NETosis primarily involves the release of mitochondrial DNA, driven predominantly by mitochondrial ROS, and is intricately associated with autoimmune diseases. Collectively, these pathways form a complex regulatory network for the production of NETs, which play a crucial role in the pathophysiology of infection, inflammation, and autoimmune diseases.

### 2.2 Components of NETs

NETs constitute a complex network composed of DNA, histones, MPO, and NE, which are released by activated neutrophils. Excessive formation of NETs has been implicated in the pathogenesis of severe vascular dysfunction and thrombosis.

#### 2.2.1 Histone

Histones H3 and H4 are recognized as highly cytotoxic elements within NETs. Research indicates that the citrullination of histone H3 is essential for the process of NETosis, and the prolonged release associated with NETosis intensifies the deleterious impact of histones on adjacent tissues (van der Linden et al., 2023). Histones have the capacity to enhance the systemic inflammatory response through selective binding to Toll-like receptors on endothelial cells. This interaction activates the NF-κB and AP-1 transcriptional pathways, subsequently inducing the release of inflammatory mediators (Yu et al., 2022). On the procoagulant front, histones facilitate the recruitment of platelets and enhance thrombin production, while also promoting platelet activation through interactions with specific platelet membrane proteins (Blanch-Ruiz et al., 2021). Furthermore, histones exhibit a strong affinity for phospholipids, enabling them to associate with endothelial cell membranes and form pores. This interaction facilitates ion influx and alters membrane potential, ultimately leading to cellular damage and apoptosis. Of greater significance, the interaction of histones with endothelial cells leads to an increase in intracellular calcium concentrations, intensifies endothelial activation and the release of Weibel-Palade bodies, promotes the secretion of von Willebrand factor (vWF), and consequently accelerates the process of thrombosis (Michels et al., 2016). *In vivo* injection experiments conducted on mice revealed that the administration of purified histone significantly elevated plasma vWF levels and exacerbated deep venous thrombosis (Brill et al., 2012).

#### 2.2.2 Chromatin DNA

The chromatin DNA within NETs serves as the reticular framework, providing the structural basis for the aggregation of platelets and erythrocytes. Additionally, it acts as a direct activator of the coagulation cascade (Rao et al., 2015). Chromatin DNA initiates the endogenous coagulation system through the activation of coagulation factor XII, while simultaneously enhancing the expression and presentation of tissue factors, thereby activating the exogenous coagulation pathway (Qi et al., 2017). In sepsis-associated coagulation disorders, NETs release elevated concentrations of DNA, which subsequently diminish plasmin production and demonstrate substantial antifibrinolytic activity, thereby impeding the natural dissolution of thrombi (Liaw et al., 2016). Research has demonstrated that circulating free DNA and MPO-DNA complexes serve as reliable biomarkers for the formation of NETs and the severity of sepsis (Pignataro et al., 2025).

#### 2.2.3 Neutrophils eastase

NE, a pivotal serine protease within NETs, exerts several procoagulant effects contributing to endothelial dysfunction and thrombosis. NE facilitates the degradation of tissue factor pathway inhibitor (TFPI) and thrombomodulin, thereby compromising the vascular endothelium’s natural anticoagulant barrier and increasing the susceptibility of the subendothelial surface to thrombotic events (Qi et al., 2017). It is noteworthy that neutrophil elastase not only facilitates coagulation but also disrupts anticoagulant mechanisms, thereby disturbing the dynamic equilibrium between the coagulation and fibrinolysis systems and leading to a procoagulant phenotype. Additionally, neutrophil elastase can directly affect endothelial cells by inducing apoptosis and structural damage, which results in the disruption of endothelial junctions and the enlargement of intercellular gaps, thereby promoting platelet adhesion and thrombosis (Baptista de Barros Ribeiro Dourado et al., 2022).

#### 2.2.4 Myeloperoxidase

MPO is a non-histone component of NETs with more than 5% content, and it is also one of the specific markers of NETosis (Pignataro et al., 2025). The myeloperoxidase-DNA complex (MPO-DNA) has the capacity to directly activate endothelial cells, thereby promoting inflammation and elevating oxidative stress levels, which culminates in endothelial dysfunction (Lin et al., 2024). MPO contributes to pro-inflammatory processes by modulating cytokines, including interleukin-1 (IL-1), interleukin-6 (IL-6), and interleukin-8 (IL-8), among others. This modulation exacerbates endothelial injury and coagulation disorders associated with sepsis. The constituents of NETs function synergistically within intricate networks. Histones activate neutrophils to produce additional NETs, thereby establishing a self-perpetuating cycle. MPO and NE collaboratively enhance coagulation by degrading anticoagulant proteins. Additionally, DNA contributes to a procoagulant milieu by activating coagulation factor XII (Blanch-Ruiz et al., 2021). In the context of sepsis, this synergistic interaction may result in immune-mediated thrombosis, microvascular obstruction, and the development of multiple organ dysfunction syndrome (MODS).

### 2.3 Release and regulation of NETs in sepsis

NETs play a crucial role in innate immunity; however, their dysregulation during sepsis can be detrimental to the host. Inflammatory cells respond to both danger-associated molecular patterns (DAMPs) and pathogen-associated molecular patterns (PAMPs), which collectively exacerbate inflammation (Cicchinelli et al., 2024).

During sepsis, PAMPs such as lipopolysaccharides (LPSs) and DAMPs like high mobility group box 1 (HMGB1) and histones activate neutrophils via pattern recognition receptors, including Toll-like receptors. Following pyroptosis, macrophages transfer mitochondria to neutrophils through microvesicles, resulting in mitochondrial dysfunction and triggering NET formation via the mitochondrial reactive oxygen species (mtROS)/gasdermin D (GSDMD) pathway. *In vivo*, microvesicles derived from pyroptotic macrophages may contribute to tissue damage, coagulation, and NET formation (Kuang et al., 2024). TREM-1 has been identified as a critical enhancer of NETosis. It synergizes with TLR4 signaling to markedly increase NET release, whereas pharmacological inhibition or genetic knockout of TREM-1 significantly reduces NETosis (Boufenzer et al., 2021). Studies indicate that sepsis patient plasma enhances platelet-neutrophil adhesion, while platelet agonists trigger TLR4-dependent NETosis, facilitating immune thrombosis and potentially causing disseminated intravascular coagulation (Carestia et al., 2016; Thomas et al., 2012).

The complement system, comprising approximately 30 serum-related proteins, is an integral component of the humoral innate immune system and demonstrates antibacterial activity. Studies have indicated that neutrophils in C3 knockout mice fail to form neutrophil extracellular traps (NETs), providing preliminary evidence that the complement system plays a role in influencing NETosis. The administration of exogenous C3 has been shown to restore NETotic capacity in these knockout mice (Yipp et al., 2012).

Complement component C3b is implicated in NET formation through either the classical or alternative pathways, and the introduction of CR1 antagonists has been observed to reduce NETosis (Palmer et al., 2016).

Furthermore, the anaphylatoxin C5a is capable of enhancing NETosis by recruiting and subsequently activating neutrophils, which leads to the upregulation of immune receptors such as Toll-like receptors (TLRs) and/or complement receptors, thereby resulting in an intensified NET response (Behnen et al., 2014; Huang et al., 2015; Wang et al., 2015). Complement proteins can initiate NET formation, which in turn can facilitate further complement activation (de Bont et al., 2019). In patients suffering from sepsis, complement proteins are nearly entirely depleted, signifying extensive immune activation that contributes to the elevated cytokine levels characteristic of the condition known as the cytokine storm (de Jong et al., 2010). Additionally, elevated levels of fibrinogen production were observed in these patients, though it remains unclear why and how the coagulation cascade is activated (de Jong et al., 2010).

## 3 Role of NETs in sepsis-induced vascular dysfunction

The endothelium constitutes the innermost cellular layer that demarcates circulating blood from the underlying tissues. It functions as a critical interface that both prevents and initiates thrombus formation, thereby contributing to the regulation of blood flow and homeostasis (Wang et al., 2018). Endothelial cells actively secrete vasoactive peptides, which modulate platelet reactivity, coagulation, fibrinolysis, and vasoconstriction, thus playing a pivotal role in thrombus formation. The release of NETs can induce endothelial damage and activation, subsequently triggering a pro-inflammatory response (Qi et al., 2017). Upon activation, endothelial cells not only initiate the coagulation cascade and recruit leukocytes to sites of endothelial injury but also enhance NET formation through the release of pro-inflammatory cytokines and the production of ROS (Gupta et al., 2010). NETs contribute to endothelial dysfunction and subsequent microcirculatory failure through various mechanisms, notably microvascular occlusion, direct endothelial damage, and immunothrombosis.

Neutrophils are recruited to sites of inflammation through the interaction between CXC chemokine ligand 1 (CXCL1) and its receptor, CXC chemokine receptor 2 (CXCR2). The process of neutrophil rolling on activated endothelial cells is facilitated by P-selection and its ligand, P-selection glycoprotein ligand 1 (PSGL-1). P-selection enhances neutrophil rolling and firm adhesion by activating integrin αLβ2 and interacting with intercellular adhesion molecule 1 (ICAM-1). The formation of NETs subsequently activates endothelial cells (ECs) through NET-associated proteases, histones, and defensins, ultimately resulting in cell death (Saffarzadeh et al., 2012). NETs can induce a proinflammatory phenotype in endothelial cells by upregulating the mRNA and protein expression of vascular cell adhesion molecule-1 (VCAM-1) and ICAM-1 (Folco et al., 2018). Furthermore, exposure to NETs leads to increased tissue factor (TF) expression and activity, fostering a procoagulant phenotype in endothelial cells (Folco et al., 2018; Song et al., 2009). This process accelerates recanalization and plasma coagulation, while treatment with anti-TF antibodies inhibits EC-induced NET-mediated coagulation (Folco et al., 2018). NETs contribute to thrombus formation both directly and indirectly. Directly, they provide a structural framework that promotes coagulation, while indirectly, they enhance the adhesion of the vascular system to other cells, potentially increasing immune cell binding and recruitment, which can further damage the vascular wall (Van Bruggen and Martinod, 2023). Furthermore, NETs facilitate thrombin production, underscoring immune thrombosis as a novel innate immune response to infection. This process results in microvascular thrombosis and supports the activity of immune cells and thrombosis-related molecules (Liu et al., 2024).

**FIGURE 2:**
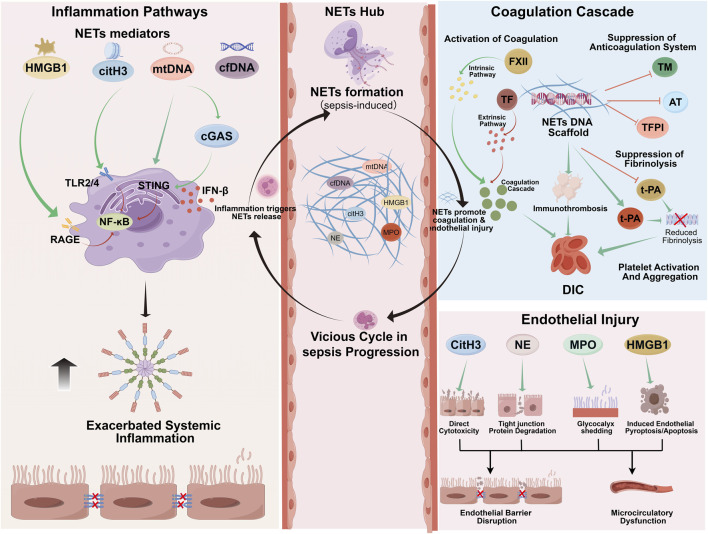
NETs serve as the central nexus in the inflammatory-coagulation-endothelial injury cycle associated with sepsis, perpetuating the condition’s progression through a closed-loop positive feedback mechanism. This cycle is characterized by the sequence: inflammation-induced NET release, followed by NET-mediated activation of coagulation disorders and endothelial injury, which in turn exacerbates inflammation. The underlying mechanism involves the sepsis microenvironment inducing neutrophil activation and subsequent NET release. This process is accompanied by the secretion and release of cfDNA, mtDNA, CitH3, HMGB1, NE, MPO, and other pivotal mediators. NETs engage the TLRP2/4-NF-κB inflammatory pathway and the cGAS-STING-IFN-β signaling pathway through their components, such as DNA and histones. Additionally, they activate the NLRP3/AIM2 inflammasome via the NETs-enriched DAMP molecule, HMGB1, which binds to the RAGE receptor to initiate the MyD88-dependent NF-κB signaling axis. This process establishes a NETs-HMGB1-RAGE-MyD88-NF-κB inflammatory positive feedback loop. Furthermore, NETs utilize their DNA network structure as a scaffold to initiate the coagulation cascade through the FXII-mediated intrinsic pathway and the TF-mediated extrinsic pathway. They also inhibit the anticoagulant system by degrading thrombomodulin and depleting antithrombin III, thereby promoting platelet activation and aggregation, which leads to immune thrombosis and potentially progresses to disseminated intravascular coagulation. Moreover, the release of histones, free DNA, and proteases results in direct cytotoxicity, degradation of endothelial tight junction proteins, and vascular endothelial cadherin (VE-cadherin). This process induces the detachment of the endothelial glycocalyx and activates the NLRP3-Caspase-1-GSDMD signaling axis, which triggers endothelial cell pyroptosis. Consequently, this damages the structural and functional integrity of the vascular endothelial barrier, leading to vascular leakage and microcirculatory dysfunction.

Overall, NETs contribute to endothelial dysfunction and subsequent microcirculatory failure through multiple intersecting mechanisms, notably microvascular occlusion, endothelial barrier disruption, and immunothrombosis (Figure 2).

### 3.1 NET-mediated endothelial injury and barrier dysfunction

#### 3.1.1 Cytotoxic effects

Sepsis is characterized by significant endothelial dysfunction, with dysregulation of endothelial cells playing a pivotal role in the complex processes that lead to organ failure (Ince et al., 2016). During sepsis, endothelial cells undergo pro-inflammatory activation, resulting in the expression of adhesion molecules that facilitate leukocyte infiltration into tissues. This infiltration ultimately contributes to organ damage through the release of proteases and oxygen-derived free radicals. Consequently, endothelial cell activation is considered a hallmark of sepsis-associated multiple organ dysfunction syndrome (MODS) (Pons et al., 2020).

NETs exert direct cytotoxic effects on vascular endothelial cells through their core components, thereby compromising the integrity of the endothelial barrier, which is critical in the pathophysiology of sepsis-associated acute lung injury (SI-ALI). Histones, which are integral components of NETs, have been shown to exhibit direct toxicity toward various cell types. For example, histones H2A, neutrophil elastase (NE), and the antimicrobial peptide LL-37 rapidly induce membrane rupture in bovine spermatozoa (Rivera-Concha et al., 2024). In the context of sepsis, NETs derived from patient neutrophils exhibit concentration-dependent cytotoxic effects on pulmonary epithelial cells (A549) (Kumar et al., 2021). Beyond direct cytotoxicity, the disruption of the vascular endothelial barrier mediated by NETs is of even greater significance. The integrity of the endothelial barrier is maintained by intercellular tight junctions and adhesion junction protein complexes. In a murine sepsis model, NETs have been shown to activate the Wnt7a/β-catenin/histone deacetylase 5 (HDAC5) signaling pathway, resulting in the downregulation of tight junction proteins such as Claudin-5, ZO-1, and Occludin, thereby compromising the function of the pulmonary microvascular endothelial barrier (Hong et al., 2026). Similarly, in a cerebral hemorrhage model, NETs disrupted the tight junctions of the blood-brain barrier by reducing the expression of ZO-1 and Occludin through the ERK/MMP9 pathway (Tang et al., 2025). This downregulation of protein expression directly contributes to a significant increase in endothelial permeability. Consequently, NETs induce endothelial barrier dysfunction and abnormally elevated vascular permeability through a dual mechanism involving both direct cytotoxic effects and the disruption of tight junction proteins (Jo and Kim, 2023).

To further dissect these pathological processes, it is essential to recognize that the individual molecular constituents of NETs mediate distinct mechanisms of endothelial injury. Specifically, the individual components of NETs drive distinct pathological processes. Cell-free DNA (cfDNA) and circulating extracellular histones (particularly H3 and H4) are considered profoundly cytotoxic components; they directly integrate into the endothelial cell membrane, leading to massive calcium influx, cellular swelling, and ultimate necrotic vascular injury (Kumar et al., 2015). Concurrently, neutrophil elastase (NE) directly contributes to barrier breakdown by proteolytically cleaving intercellular junctional proteins, notably VE-cadherin, thereby severely increasing paracellular permeability (Ushakumari et al., 2022). Furthermore, granular enzymes such as myeloperoxidase (MPO) act synergistically with NE to disrupt junction integrity, exacerbate oxidative stress, and induce cytoskeletal contraction, leading to severe intercellular gaps and endothelial hyperpermeability (Ma et al., 2019).

#### 3.1.2 Impairment of endothelial regeneration and repair

In the context of sepsis, vascular endothelial cell injury is persistent, and the capacity for repair is crucial for maintaining organ function. However, excessive formation and accumulation of NETs not only initiate damage but also severely impair the regenerative and reparative potential of endothelial cells, thereby hindering recovery post-injury. The underlying mechanism of this suppression involves interference with key cell cycle regulatory pathways. Research has demonstrated that excessive accumulation of NETs adversely affects the proliferative capacity of pulmonary endothelial cells. This phenomenon is mechanistically linked to the inhibition of the Polo-like kinase 1 (PLK1) signaling pathway (Jo and Kim, 2023). PLK1 is a pivotal kinase involved in the regulation of the mitotic process, essential for the transition of cells from the G2 phase to the M phase, thereby facilitating cell division. The inhibition of PLK1 signaling by NETs results in the arrest of endothelial cells at the G2/M phase, thereby obstructing their progression into the division phase. This arrest fundamentally impedes the critical processes of endothelial cell regeneration and repair, which are necessary following vascular injury. Such cell cycle arrest hinders the endothelium’s ability to proliferate and repair defects caused by factors such as NETs, leading to prolonged impairment of barrier function and exacerbating persistent tissue edema and inflammation. An effective intervention strategy involves degrading the DNA backbone of NETs using deoxyribonuclease I (DNase I). In the context of sepsis-associated acute lung injury, the degradation of NETs has been shown to reduce pulmonary inflammation and facilitate the recovery of endothelial function. In a sepsis-associated encephalopathy (SAE) model, the inhibition of NET formation using the PAD4 inhibitor GSK484 not only mitigated neuroinflammation but also enhanced Occludin expression through the Wnt3/β-catenin/TCF4 signaling pathway. This process facilitated the assembly of the vascular endothelial cell (VE-cadherin)/β-catenin/vimentin (VCL) complex, thereby strengthening blood-brain barrier integrity and improving cognitive function (Yue et al., 2025). Overall, NETs compromise endothelial repair capacity by inhibiting critical signaling pathways that regulate endothelial proliferation and by disrupting junctional complexes essential for maintaining barrier integrity. Consequently, DNase I or PAD4 inhibitors may represent viable therapeutic strategies for preserving vascular homeostasis and promoting tissue repair.

#### 3.1.3 Disruption of the endothelial glycocalyx

The vascular endothelial glycocalyx is a complex, web-like structure lining the luminal surface of vascular endothelial cells. Composed of glycoproteins, proteoglycans, and glycosaminoglycans, it plays a paramount role in maintaining vascular barrier integrity, regulating blood flow, and mediating anticoagulant and anti-inflammatory functions (Dancy et al., 2024). During the pathogenesis of sepsis, the hyperactivation of neutrophils and the subsequent release of neutrophil extracellular traps (NETs) inflict direct and severe damage upon the endothelial glycocalyx. Various enzymes embedded within NETs, particularly neutrophil elastase and matrix metalloproteinase-9, can specifically degrade key constituents of the glycocalyx, such as heparan sulfate and hyaluronic acid (Zhang et al., 2023). As heparan sulfate is the core molecule responsible for maintaining the negative charge and anticoagulant properties of the glycocalyx, its degradation directly compromises the antithrombotic capacity of endothelial cells. Concurrently, the shedding of hyaluronic acid disrupts the physical barrier of the glycocalyx, leading to a pronounced increase in vascular permeability. This NET-mediated degradation of the glycocalyx represents a central mechanism underlying vascular endothelial dysfunction and microcirculatory failure in sepsis. The disruption of the glycocalyx triggers a cascade of detrimental events, exacerbating the vascular pathologies associated with sepsis. An intact glycocalyx utilizes its dense array of surface negative charges to form an electrostatic repulsive barrier, effectively preventing the non-specific adhesion of leukocytes and platelets to endothelial cells. Following the NET-induced degradation of its components, the negative charge on the endothelial surface is markedly diminished, resulting in the loss of this electrostatic barrier (Iba et al., 2025). The direct consequence is the enhanced adhesion of circulating leukocytes and platelets to the denuded endothelium, which initiates and amplifies local inflammatory responses and thrombotic processes. This aberrant adhesion further activates endothelial cells, shifting their phenotype toward a pro-inflammatory and procoagulant state, thereby creating a self-amplifying vicious cycle (Zhang et al., 2023). Ultimately, microvascular leukocyte stasis, platelet aggregation, and microthrombus formation lead to tissue hypoperfusion, ischemia, and hypoxia, serving as a critical mechanism driving the progression of sepsis to multiple organ dysfunction.

### 3.2 Role of NETs in sepsis-associated immunothrombosis

#### 3.2.1 Activation of the coagulation cascade and inhibition of anticoagulant pathways

NETs are integral to the coagulopathy observed in sepsis, as they activate the coagulation system and inhibit physiological anticoagulation through various mechanisms. At the molecular level, each NET component plays a highly specialized role in establishing a procoagulant microenvironment. The extracellular DNA web provides a dense, strongly electronegative structural scaffold that acts as a potent surface for the autoactivation of Factor XII, thereby directly initiating the intrinsic contact pathway of coagulation (Noubouossie et al., 2019). Research suggests that the DNA framework of NETs inherently exhibits procoagulant properties, significantly enhancing thrombin generation (Whitefoot-Keliin et al., 2024). This procoagulant effect is further intensified when NETs electrostatically interact with neutrophil-derived extracellular vesicles (EVs) (Whitefoot-Keliin et al., 2024). These vesicles, which are released in response to bacterial stimuli such as *Staphylococcus aureus*, *Staphylococcus* epidermidis, *Escherichia coli*, and *Pseudomonas aeruginosa*, contain surface-bound DNA that initiates and amplifies the coagulation cascade in a DNA-dependent manner, resulting in significantly increased thrombin generation (Whitefoot-Keliin et al., 2024). Additionally, histones present on NETs are crucial in disrupting normal anticoagulant pathways. Circulating histones, functioning as damage-associated molecular patterns, bind to and inactivate essential anticoagulants such as thrombomodulin and tissue factor pathway inhibitor (Li et al., 2021). Moreover, cell-derived free histones directly initiate coagulation by inducing the expression of tissue factor on endothelial cells and monocytes (Yong et al., 2023). In addition to activating tissue factor, histones directly bind to prothrombin to accelerate its cleavage into thrombin and induce profound platelet activation and aggregation via TLR2 and TLR4 pathways, thereby potently amplifying the coagulation cascade (Semeraro et al., 2011). Furthermore, NET-associated serine proteases actively dismantle physiological anticoagulant mechanisms. Specifically, NE and MPO synergistically bind and proteolytically cleave tissue factor pathway inhibitor (TFPI), effectively unleashing the extrinsic coagulation pathway mediated by tissue factor (Martinod and Wagner, 2014). This reciprocal coupling of coagulation and innate immunity via neutrophil serine proteases profoundly accelerates uncontrolled fibrin formation (Massberg et al., 2010).

In recent years, platelets have been increasingly recognized as mature innate immune cells with the capability to detect and neutralize invading pathogens. Consequently, platelets serve as a critical interface between the immune system and coagulation processes (Zucoloto and Jenne, 2019). They are essential in hemostasis and thrombosis (van der Meijden and Heemskerk, 2019). In the context of severe bacterial sepsis, research suggests that platelets function as sensors of systemic infection by binding to TLR4 on their membranes, which facilitates the recruitment of isolated neutrophils. Upon platelet stimulation, neutrophils significantly increase the surface area of NETs generated *in vitro* (Schattner et al., 2020). These NETs can be released into the vascular lumen, adhere to the vascular wall, and initiate prothrombotic and procoagulant responses through TLR4 interaction (Semeraro et al., 2011).

Further studies suggest that NETs activate platelets and carry tissue factor, thereby initiating and amplifying the coagulation cascade. The formation of NETs complexes with platelets, enhancing immune and coagulation responses through positive feedback loops (Chen et al., 2025). In sepsis, NETs also contribute to endothelial cell injury, leading to substantial tissue factor production and exacerbating the dysregulation of inflammation and coagulation (Zhu S. et al., 2023). In summary, NETs constitute a potent procoagulant network by providing procoagulant surfaces (cfDNA), inactivating anticoagulants (Histones, NE, MPO), and activating platelets and tissue factor, representing a key mechanism of sepsis-induced coagulopathy (SIC) (Chen et al., 2022).

At the molecular level, the extracellular DNA scaffold of NETs acts as a strongly electronegative surface that activates the intrinsic coagulation pathway via Factor XII. Concurrently, NE and MPO interact with tissue factor pathway inhibitor (TFPI), cleaving and inactivating it, which unchecked leads to rampant extrinsic pathway activation.

#### 3.2.2 Resistance to fibrinolysis and formation of microthrombus scaffolds

The interplay between neutrophil activation, the release of NETs, and their interactions with the coagulation system—collectively referred to as immunothrombosis—functions as a protective mechanism against bacterial dissemination during localized infection (Kessenbrock et al., 2009; Delvaeye and Conway, 2009). While NETs play a crucial role in immune thrombosis, excessive formation of NETs can result in extensive coagulation disorders and subsequent organ failure (Guo et al., 2025). Notably, NET-induced thrombosis is a common pathogenic feature extending beyond sepsis, frequently observed in various autoimmune diseases as well (Fuchs et al., 2010). To resolve NET-induced thrombi within the circulatory system, NETs are degraded by host deoxyribonucleases (DNases), which constitute a critical regulatory mechanism in immune thrombosis (Jiménez-Alcázar et al., 2017). Consequently, the administration of exogenous DNase is being explored as a potential therapeutic strategy for managing immune thrombosis (Jiménez-Alcázar et al., 2017). The interaction between platelets and NETs further facilitates immune thrombosis (McDonald et al., 2017), with research suggesting that NETs may serve as pre-thrombotic lesions, providing a scaffold for thrombus formation and enhancing thrombus stability and growth (de Boer et al., 2013).

NETs not only initiate coagulation but also play a pivotal role in sepsis microvascular thrombosis by counteracting the fibrinolytic system and constructing stable microthrombus scaffolds. First, histones and NE within NETs can directly interfere with the fibrinolytic system, stabilizing microthrombi. Histones inhibit plasminogen activation and protect fibrin from fibrinolytic degradation (Zhou et al., 2022). Furthermore, NETs form robust “immune thrombi” by tightly interweaving with platelets, red blood cells, and fibrin around their DNA scaffold core, thereby occluding microvessels (Aklilu et al., 2025). This structure provides a physical scaffold for thrombi, with the reticular architecture of NETs effectively trapping platelets and red blood cells while intertwining with fibrin to form microthrombi that are difficult to clear (Iba et al., 2025). In sepsis-associated acute lung injury, NETs induce endothelial cell damage via the STING pathway and generate excessive tissue factor, amplifying the coagulation cascade and promoting immune thrombus formation (Zhu S. et al., 2023). While immune thrombi represent a physiological defense mechanism, their abnormal activation in sepsis leads to microvascular thrombosis, organ ischemia, and potential progression to disseminated intravascular coagulation (DIC) (Aklilu et al., 2025). As a pivotal junction between inflammation and coagulation, excessive NET formation redirects the immune response toward pathological thromboinflammation, ultimately causing tissue hypoperfusion and multiple organ dysfunction (Zhang et al., 2023).

## 4 NET-related treatment

Sepsis is a critical infectious response that may cause immune imbalance, vascular issues, and organ failure. New approaches are needed to identify diagnostic and therapeutic targets (Sorrentino et al., 2022). NETs can both fight pathogens and intensify tissue and organ damage, while also amplifying the body’s inflammatory response. Direct intervention in the formation and clearance of NETs represents a pivotal therapeutic strategy for addressing vascular dysfunction in sepsis. Excessive formation of NETs and their impaired clearance are significant mechanisms contributing to endothelial injury and immune-mediated thrombosis. Therefore, targeting the reduction of NETs release or production and promoting their degradation may offer a promising research avenue for disease treatment. The clinical use of drugs specifically targeting NETs remains underdeveloped, although research on the mechanisms regulating NET inhibition is a key focus in treatment studies.

Accumulating direct evidence from *in vivo* sepsis models strongly supports that inhibiting NET formation significantly improves both vascular function and overall survival rates. In murine models, the administration of NET-degrading enzymes, such as DNase I, effectively removes NETs, decreases platelet aggregation, reduces intravascular coagulation, and notably improves microvascular perfusion. Furthermore, directly targeting NET production via PAD4 inhibitors, such as Cl-amidine, or utilizing DNase I has been explicitly shown to suppress NET formation, attenuate systemic inflammation, and significantly improve survival outcomes in septic mice. While these preclinical studies provide robust proof-of-concept that abrogating NETosis preserves endothelial integrity and prevents sepsis progression, direct clinical evidence demonstrating mortality reduction in human sepsis patients remains limited, highlighting a critical translational gap that future clinical trials must address.

### 4.1 Directly interfering with the formation and clearance of NETs

Direct intervention in the formation and clearance of NETs represents a pivotal therapeutic strategy for addressing vascular dysfunction in sepsis. Excessive formation of NETs and their impaired clearance are significant mechanisms contributing to endothelial injury and immune-mediated thrombosis. Research indicates that inhibiting NETosis or enhancing the degradation of existing NETs can effectively alleviate organ injury associated with sepsis. For example, in an experimental model of neonatal infectious peritonitis, administration of a neonatal NET inhibitor (nNIF), the PAD4 inhibitor Cl-amidine, or the degradative enzyme DNase I significantly reduced intraperitoneal NET formation and inflammatory cytokine levels, while also improving survival rates (Denorme et al., 2023).

This evidence suggests that directly targeting the pathways involved in NET generation and clearance offers substantial therapeutic potential. In a model of sepsis-induced cardiomyopathy, inhibiting NET formation—using agents such as the PAD4 inhibitor Cl-amidine or the neutrophil elastase inhibitor Sivelestat—or degrading NETs with DNase I, resulted in reduced myocardial inflammation and apoptosis, thereby partially preserving cardiac contractility (Xue et al., 2026). These interventions alleviate the cytotoxic and prothrombotic effects of NETs on the vascular endothelium by decreasing circulating NET levels. Moreover, research suggests that impaired NET clearance in sepsis may be associated with age. Consequently, enhancing NET clearance mechanisms—such as activation of the AMP-activated protein kinase (AMPK) pathway or the administration of exogenous DNase—could constitute innovative strategies to improve outcomes in elderly sepsis patients (Table 1).

**TABLE 1:** Potential therapeutic agents targeting NETs in sepsis.

Drugs	Mechanism	Preclinical/Clinical evidence	Development status	References
DNase I	Cleaves extracellular DNA scaffold of NETs	Reduces immune thrombosis and organ injury in animal models (e.g., neonatal infectious peritonitis, pancreatitis, SAE)	Experimental in sepsis; clinically used for cystic fibrosis	Denorme et al. (2023), Xue et al. (2026), Li et al. (2026)
Cl-amidine/GSK484	PAD4 inhibition, blocks chromatin decondensation	Improves survival, reduces microvascular thrombosis, and preserves blood-brain barrier integrity in murine sepsis/SAE.	Preclinical	Yue et al. (2025), Biron et al. (2017)
PAD2 inhibitors	PAD2 inhibition	Decreased NET formation, leading to enhanced survival and organ function in sepsis patients	Preclinical/experimental	Huang et al. (2020)
Liang-Ge decoction	Inhibits PAD4-mediated NET formation	Ameliorates coagulation dysfunction in sepsis rats	Preclinical	He et al. (2023)
LR12 peptide	TREM-1 inhibitor, blocks amplification of TLR4 signaling	Prevents NET release and protects vascular reactivity in septic shock	Clinical Trials (Phase II/III for septic shock)	Boufenzer et al. (2021)
TAK-242	Specific TLR4 antagonist/blocker	Arrests downstream NF-κB activation and subsequent NET formation, mitigates systemic inflammation	Preclinical/clinical evaluation	Ii et al. (2006)
Sivelestat	Neutrophil Elastase (NE) specific competitive inhibitor	Preserves cardiac contractility, attenuates microvascular leakage, and reduces multiple organ injury	Clinically approved for ARDS in some countries; under sepsis evaluation	Hayakawa et al. (2010), Hagiwara et al. (2009)
Recombinant human soluble thrombomodulin (rTM)	Neutralizes DAMPs (histones, HMGB1), generates activated protein C (APC), anticoagulant	Inhibits NETs, reduces cytokine storm, improves microvascular perfusion	Clinically approved for DIC in some countries	Suzuki, 2025; Kato et al. (2021), Iba et al. (2014)
Histone-neutralizing antibodies	Specific binding and neutralization of extracellular histone toxicity	Alleviates organ damage, preserves vascular barrier function, and attenuates subsequent inflammatory cascades	Preclinical	Lei et al. (2024), Chen et al. (2024)
Disulfiram	GSDMD inhibitor, blocks pore formation	Abrogates NETosis, prevents multiple organ dysfunction, improves survival in severe sepsis models	FDA-approved for alcoholism; repositioning for sepsis	Silva et al. (2021)
(2S)-OMPT	Lysophosphatidic acid receptor 3 (LPA3) agonist	Suppresses NET production and associated microvascular thrombosis	Preclinical	Pei et al. (2022)

#### 4.1.1 DNase I

DNase I enzymatically hydrolyzes extracellular DNA and functions as a pivotal enzyme in the degradation of NETs, offering significant therapeutic potential in mitigating NETs-mediated pathological damage. In the context of sepsis, the DNA backbone of NETs serves as their primary structural component, underpinning their role in mediating cytotoxicity, prothrombotic effects, and exacerbated inflammation. By cleaving this DNA scaffold, DNase I effectively disrupts the NETs network, thereby attenuating its pathological consequences. Numerous studies have substantiated the protective role of DNase I in sepsis models. For instance, in experimental neonatal infectious peritonitis, DNase I treatment resulted in reduced intraperitoneal NET levels and enhanced survival rates when administered in conjunction with antibiotics (Denorme et al., 2023). Similarly, in a sepsis-induced cardiomyopathy model, DNase I facilitated the degradation of NETs, leading to decreased myocardial inflammation and apoptosis (Xue et al., 2026). In the context of severe acute pancreatitis, which serves as a model for systemic inflammation, DNase I has been shown to synergistically degrade NETs when used in conjunction with N-acetylcysteine (NAC). This combination mitigates pancreatic and pulmonary injury by inhibiting the NF-κB/CXCL3 signaling pathway (Li et al., 2026). Beyond its degradative capabilities, DNase I also modulates NET function. For example, NETs have been found to degrade the T-cell chemokine CXCL11, thereby impeding the chemotactic migration of activated T cells—a process that can be reversed with DNase I treatment (Tamura et al., 2023). However, the application of DNase I must be approached with caution, as excessive degradation of NETs could undermine their early-stage defensive role in trapping and neutralizing pathogens. Research suggests that the premature use of DNase I to eliminate NETs in sepsis may exacerbate inflammatory responses and lead to increased organ damage (Duan et al., 2021). Consequently, the timing and dosage of DNase I therapy require meticulous calibration. The objective is to selectively clear pathological, excessive NETs while preserving their essential immune defense functions. This strategy aims to reduce vascular endothelial injury and immune thrombosis, thereby fostering an environment conducive to endothelial repair.

Furthermore, immune cells release DNase 1-like 3, which specifically targets DNA-protein complexes, synergizing with DNase 1 to protect the endothelium during sepsis (Jiménez-Alcázar et al., 2017; Sisirak et al., 2016).

#### 4.1.2 PAD4 inhibitors

Overexpression of PAD4 causes notable vascular damage by enhancing NET release and increasing ICAM-1 and Vasopressin-activated calcium-mobilizing (VACM-1) expression on endothelial cells (Folco et al., 2018). Consequently, PAD4 inhibitors can inhibit NET formation and prevent endothelial cell dysfunction. PAD4 could be a potential therapeutic target for acute lung injury caused by sepsis (Folco et al., 2018). PAD4 knockout mice demonstrated improved survival and reduced organ dysfunction and sepsis progression in a sepsis model (Assinger et al., 2011). Liang-Ge (LG) decoction ameliorates coagulation dysfunction in sepsis rats by inhibiting PAD4-mediated NET formation (He et al., 2023). In addition, one study showed elevated levels of PAD2 protein in patients with sepsis. PAD2 inhibitors were shown to decrease NET formation, leading to enhanced survival and organ function in sepsis patients (Huang et al., 2020). In a mouse sepsis model, the PAD4 inhibitor Cl-amidine effectively suppressed NET formation and improved survival rates (Biron et al., 2017). However, studies indicate that the formation of sepsis-induced NETs is regulated by distinct stimulus-dependent pathways, which may operate independently of the typical PAD4 and GSDMD mechanisms (Englert et al., 2025).

### 4.2 Therapeutic targeting of upstream regulatory mechanisms

A comprehensive strategy to address sepsis-induced vascular dysfunction involves targeting the upstream regulatory mechanisms associated with NETs. This strategy extends beyond the NETs themselves to include the modulation of intracellular signaling pathways that govern their formation and intercellular interactions prior to NET deployment. Upstream regulation focuses on critical molecular regulators of neutrophil activation, migration, and NETosis, such as the activity of specific kinases, surface receptors, and intercellular cross-talk. Research suggests that NET formation in sepsis is modulated by a complex regulatory network. For instance, S100A8/A9 activates platelets via TLR4, resulting in GSDMD-dependent platelet pyroptosis. The subsequent release of oxidized mitochondrial DNA (ox-mtDNA) further promotes NET formation, which in turn releases S100A8/A9, thereby establishing a positive feedback loop that perpetuates inflammation (Tang and Hwa, 2022). Targeting key nodes within these circuits—such as suppressing excessive platelet activation or blocking inflammatory amplifiers—may effectively suppress the excessive production of NETs at their origin, providing a robust upstream defense to protect endothelial integrity (Guan et al., 2025).

#### 4.2.1 TREM-1 inhibitors

The TREM-1 receptor is crucial in amplifying inflammatory responses, as its activation enhances TLR4-mediated signaling pathways, thereby promoting neutrophil activation and NETosis. Direct experimental evidence demonstrates that TREM-1 acts as a potent driver of NET release in human and murine neutrophils. In the context of sepsis, overexpression of TREM-1 is associated with increased disease severity and poor prognosis. Studies have shown that pharmacological inhibition of TREM-1 using the synthetic peptide LR12, or its genetic ablation, significantly decreases NETosis *in vitro* and effectively protects against endothelial dysfunction in experimental septic shock models *in vivo* (Boufenzer et al., 2021). By disrupting this amplification loop, TREM-1 inhibitors prevent neutrophil hyperactivation and shield endothelial cells from cytotoxic damage inflicted by NET-associated histones and granular proteins, making it a promising therapeutic target. Additionally, S100A8/A9 derived from septic conditions triggers platelet pyroptosis via the TLR4-ROS-NLRP3-caspase-1 pathway, with the resultant ox-mtDNA release further promoting NET formation (Su et al., 2022). These studies indirectly highlight the significance of targeting upstream inflammatory amplifiers, such as the TLR4 pathway, to regulate NETosis. As a synergistic enhancer of TLR4 signaling, TREM-1 inhibitors are theoretically capable of disrupting this amplification loop, thereby mitigating neutrophil hyperactivation and NET release. This intervention could potentially shield endothelial cells from cytotoxic damage inflicted by NET-associated histones and granular proteins. Future research should aim to directly assess the specific impact of TREM-1 inhibitors on reducing NET formation, improving endothelial function, and enhancing outcomes in sepsis models.

#### 4.2.2 Platelet and exosome targeted therapy

Inhibition of IκB kinase (IKK) to attenuate platelet-derived exosome secretion, alongside neutralization of HMGB1 and specific microRNAs within exosomes, can effectively impede excessive NET formation. Platelets and their secreted exosomes are crucial in the pathogenesis of sepsis-induced immunothrombosis and the induction of NETs. Activated platelets not only engage directly with neutrophils but also modulate neutrophil function at a distance by releasing exosomes enriched with bioactive molecules, thereby facilitating NETosis. Consequently, targeting platelet activation or their exosome secretion emerges as a promising strategy to mitigate NET overproduction. Research indicates that in sepsis, platelets undergo GSDMD-dependent pyroptosis following TLR4-mediated detection of S100A8/A9, leading to the release of ox-mtDNA, a potent NET inducer (Su et al., 2022). This process is characterized by intricate intracellular signaling pathways. Inhibition of IKK, a critical upstream component of the NF-κB signaling pathway, may diminish platelet overactivation and inflammatory cytokine production, thereby indirectly curtailing their capacity to enhance NET formation. Furthermore, platelet-derived exosomes function as crucial vectors for the transport of signaling molecules, including HMGB1 and specific microRNAs (miRNAs). HMGB1, recognized as a significant DAMP, is a potent inducer of NETosis. Empirical evidence supports the existence of a positive feedback loop involving NET formation and oxidative stress/NLRP3 inflammasome activation, with HMGB1 acting as a pivotal mediator (Dehdashtian and Caricchio, 2025). Consequently, neutralizing HMGB1 within exosomes or inhibiting its receptors, such as RAGE, through the use of antibodies or inhibitors, can effectively diminish its neutrophil-activating signals. Similarly, specific pro-inflammatory miRNAs contained within exosomes may modulate gene expression related to NETosis in neutrophils. The development of antisense oligonucleotides (ASOs) or miRNA inhibitors (antagomirs) to neutralize these miRNAs could suppress NET formation at the epigenetic or post-transcriptional level. In conclusion, therapeutic strategies targeting the platelet-exosome axis—either by inhibiting their secretion or by neutralizing their deleterious contents—present innovative approaches to mitigate excessive NET formation at the level of intercellular communication.

#### 4.2.3 TLR4 inhibitors

The interaction between TLR4 and LPS is associated with sepsis development, and inhibiting TLR4 improves sepsis prognosis. TLR4 on platelets is also involved in the induction of NETs. Therefore, TLR4 blockers may slow the progression of sepsis by reducing the production of NETs. Tissue and organ damage from sepsis is due to an excessive inflammatory response, immune system dysfunction, and coagulation issues. In sepsis, DAMP molecules are pivotal in causing excessive inflammation. Previous research identified NMI as a novel DAMP molecule that intensifies sepsis-related inflammation by interacting with TLR4 on macrophages, activating the NF-κB pathway, and inducing pro-inflammatory cytokine release (Zeng et al., 2024). The study found that TLR 4 −/− mice, unlike the wild-type controls, showed no rise in thrombus formation and had notably reduced circulating ICAM-1 levels. Consequently, inhibiting TLR 4 may enhance clinical outcomes for sepsis patients (Obi et al., 2017; Schattner, 2019). Notably, in experimental endotoxemia, the specific TLR4 antagonist TAK-242 mitigates systemic inflammation by binding selectively to the intracellular domain of TLR4, thereby arresting downstream NF-κB activation and subsequent NET formation (Ii et al., 2006). Consequently, TLR 4 inhibitors present a promising upstream therapeutic intervention to prevent sepsis-induced microvascular thrombosis. The TLR4 blocker shows promise as a novel therapeutic target for sepsis prevention and treatment, but requires further validation through additional basic and clinical research.

### 4.3 Neutralization of downstream pathological effectors

Conversely to upstream regulation, downstream interventions aim to neutralize the toxic components already released by NETs into the extracellular milieu. These include free histones, HMGB1, and granule proteases, which can directly damage endothelial cells, disrupt adherens junctions, and exacerbate systemic coagulation. Directly neutralizing these pathological effectors can shield the vascular endothelium from established NET-driven cytotoxicity.

#### 4.3.1 Recombinant human soluble thrombomodulin

Recombinant human soluble thrombomodulin (rTM) is a therapeutic protein with potent anticoagulant and anti-inflammatory properties, currently utilized in treating sepsis-associated disseminated intravascular coagulation (DIC) (Suzuki, 2025). In experimental sepsis models, rTM exerts direct organ-protective effects by inhibiting NET formation and neutralizing DAMPs (Kato et al., 2021). Specifically, rTM binds to and neutralizes extracellular histones and HMGB1, effectively severing the positive feedback loop of NETosis and inflammation (Iba et al., 2014). Furthermore, its ability to generate activated protein C (APC) limits thrombin generation, thereby indirectly attenuating platelet-mediated neutrophil activation (Martos et al., 2020). These multi-pathway interventions provide robust evidence supporting rTM as a viable strategy to suppress the NET-driven cytokine storm and improve microvascular perfusion in sepsis.

#### 4.3.2 Histone-neutralizing antibodies

Histones are integral toxic components of NETs. During the formation of NETs, nuclear histones, such as H3 and H4, undergo citrullination and are subsequently released into the extracellular milieu alongside DNA. These extracellular histones exhibit significant cytotoxic properties, directly compromising the integrity of endothelial cell membranes, inducing cell death, and activating inflammatory and coagulation pathways. As key effector molecules, they contribute to vascular dysfunction in sepsis. Consequently, the use of histone-neutralizing antibodies to specifically bind and neutralize extracellular histone toxicity constitutes a protective strategy aimed at mitigating the downstream effects of NET-mediated injury. In systemic inflammatory conditions such as sepsis, elevated levels of circulating histones are closely associated with organ damage and increased mortality. Histones inflict damage on endothelial cells by disrupting membrane integrity, inducing calcium influx, promoting the production of ROS, and activating TLR signaling pathways, including TLR2 and TLR4. Inhibition of these pathological processes can be achieved through the use of histone-specific antibodies. For example, in relevant disease models, anti-histone antibodies have been shown to alleviate organ damage caused by endotoxemia or ischemia/reperfusion injury. Although the referenced studies do not explicitly address the application of histone antibodies in sepsis, they highlight the critical role of NET components, including histones, in mediating inflammation and thrombosis (Lei et al., 2024). Additionally, research suggests that NETs activate intracellular DNA sensors such as cGAS and TLR9 via their DNA-histone complexes, thereby promoting NF-κB-dependent autoimmunity and inflammation (Chen et al., 2024). Neutralizing histones may not only diminish their direct cytotoxic effects but also disrupt the recognition of NETs-DNA complexes by immune cells, thus attenuating subsequent inflammatory cascades. Therefore, the development of highly efficient and specific neutralizing antibodies or antibody fragments targeting histones represents a promising therapeutic strategy to protect the compromised vascular endothelium in sepsis. This approach could help preserve vascular barrier function, reduce microthrombus formation, and mitigate organ hypoperfusion.

#### 4.3.3 Neutrophil elastase inhibitors

In addition to histones and HMGB1, granule proteases mobilized onto the NET scaffold—particularly neutrophil elastase (NE)—remain highly proteolytically active in the extracellular space. As a critical downstream effector, NE directly cleaves intercellular junctional proteins (such as VE-cadherin) and severely degrades the endothelial glycocalyx, driving vascular hyperpermeability and edema. Consequently, the pharmacological inhibition of NE constitutes a vital downstream strategy to protect endothelial barrier integrity. Sivelestat, a specific and competitive inhibitor of NE, has been shown to effectively neutralize the proteolytic damage inflicted by NET-associated elastase (Hayakawa et al., 2010). In preclinical models of sepsis and acute respiratory distress syndrome (ARDS), the administration of Sivelestat significantly attenuated microvascular leakage, preserved endothelial contractility, and reduced multiple organ injury (Hagiwara et al., 2009). While Sivelestat is clinically approved for ARDS in certain countries, its precise efficacy in broadly reversing NET-driven systemic endothelial dysfunction in sepsis warrants further large-scale clinical validation.

### 4.4 Other treatments

ROS act as central second messengers in the induction of NETosis. Consequently, targeted antioxidant therapies can effectively scavenge excess ROS, directly hindering the chromatin decondensation step of NET formation and limiting oxidative distress in the endothelium (Nardi et al., 2014). Furthermore, the pore-forming protein GSDMD plays an executive role in NET release. Disulfiram, an FDA-approved drug that specifically inhibits GSDMD, has been shown to successfully abrogate NET formation in septic mice, thereby preventing multiple organ dysfunction and significantly improving survival rates (Silva et al., 2021). Finally, lysophosphatidic acid (LPA) signaling modulates neutrophil behavior. The specific LPA receptor 3 (LPA3) agonist (2S)-OMPT exhibits a crucial protective role in sepsis by diminishing NET production and associated microvascular thrombosis, presenting a novel lipid-mediated target for therapeutic intervention (Pei et al., 2022).

In summary, therapeutic strategies targeting the NETosis pathway—ranging from upstream regulation of neutrophil activation (e.g., TREM-1 and TLR4 blockers) to the direct inhibition of chromatin decondensation (e.g., PAD4 inhibitors) and the degradation of extracellular DNA scaffolds (e.g., DNase I)—have demonstrated profound efficacy in in vivo experiments. These targeted interventions consistently mitigate endothelial injury, prevent immunothrombosis, and improve survival in diverse animal models of sepsis. Despite these promising preclinical results, the transition to clinical application remains in its infancy. Except for agents like recombinant human soluble thrombomodulin (rTM) and Sivelestat, which have seen clinical application for related coagulopathies or respiratory distress, most NET-specific inhibitors have not yet advanced to large-scale Phase II/III clinical trials for sepsis. Consequently, accelerating the clinical validation of these targeted therapies is essential to determine their safety and efficacy in heterogeneous septic populations.

## 5 Conclusion

Sepsis remains a leading cause of global mortality, characterized by profound vascular dysfunction and organ failure. While neutrophils are essential for infection control, their dysregulated release of NETs forms a self-amplifying pathological network connecting inflammation, coagulation, and endothelial injury. This review synthesizes key mechanisms, highlighting how specific NET components—such as histones, DNA, and proteases—directly disrupt endothelial barriers and initiate microvascular immunothrombosis. Despite promising preclinical evidence for NET-targeted therapies like DNase I, PAD4 inhibitors, and GSDMD blockers, several critical knowledge gaps remain. Firstly, the current understanding of the regulatory network involved in the formation of NETs remains incomplete. Existing research has predominantly concentrated on individual cell types or specific stimulators, whereas sepsis represents a complex pathological condition characterized by the involvement of multiple cell types and pathways. Consequently, further investigation is necessary to identify the key molecules that regulate NET formation, optimize the precise timing and threshold for NET inhibition, prevent pathogenic tissue damage, and provide targets for the development of precise intervention strategies. Secondly, there exists a significant gap in our understanding of the mechanisms by which pathogens evade NETs and subsequently exacerbate vascular damage. Future research should aim to identify NET escape factors associated with various pathogens and elucidate their specific mechanisms. Lastly, there is an urgent need for clinical trials to determine whether circulating NET biomarkers can effectively guide personalized therapy and serve as reliable indicators for dynamically monitoring NET load and activity in patients. Emphasis should be placed on the development of highly selective and co-targeted therapies, the construction of multidimensional prognostic models, and the ultimate translation of these pathophysiological insights into effective clinical interventions.
